# Mapping and modulating brain dynamics in aging and neurodegeneration with electroencephalography and integrated neurotechnologies in primates

**DOI:** 10.3389/fnins.2026.1689222

**Published:** 2026-08-25

**Authors:** Ankur Gupta, Yulia Dembitskaya, Anton Pushkarev, Alexander Popov

**Affiliations:** 1 University Bordeaux, CNRS, IMN, UMR 5293, Bordeaux, France; 2 Shemyakin Ovchinnikov Institute of Bioorganic Chemistry, Russian Academy of Sciences, Moscow, Russia; 3 Kurchatov Medical Primatology Center, NRC “Kurchatov Institute”, Sochi, Russia

**Keywords:** aging, electrocorticography, electroencephalography, neurodegeneration, neuromodulation, neurotechnologies, non-human primates (NHPs)

## Abstract

Non-human primates (NHPs) provide an essential translational platform for investigating neural mechanisms underlying brain aging and neurodegenerative diseases. Their neuroanatomical organization, cortical complexity, and cognitive capacities share close parallels with those of humans, enabling a degree of experimental precision that rodent models cannot achieve. Electroencephalography (EEG) offers a non-invasive method for monitoring neuronal population activity with high temporal resolution, capturing oscillatory patterns and connectivity changes that emerge over the course of aging and in pathological states. In humans, such measures have revealed alterations in dominant frequency bands, network synchrony, and event-related potentials that serve as candidate biomarkers for cognitive decline. Extending EEG to NHP models permits the direct examination of mechanistic hypotheses and assessment of therapeutic interventions in a brain closely resembling that of humans. We emphasize study-level examples and quantitative anchors, including frequency-band ranges and the direction of EEG changes across aging and neurodegenerative disease models. Recent work has expanded EEG applications in NHPs to longitudinal studies, invasive/non-invasive signal comparisons, and integration with other modalities, including magnetoencephalography (MEG), functional magnetic resonance imaging (fMRI), functional near-infrared spectroscopy (fNIRS), and invasive electrophysiology. Furthermore, pairing EEG with neuromodulation techniques such as transcranial magnetic stimulation (TMS), transcranial direct and alternating current stimulation (tDCS, tACS), temporal interference (TI) stimulation, and transcranial focused ultrasound (tFUS) offers a framework for causally probing and modifying network function. This review synthesizes EEG methodologies in NHPs, examines their applications to models of normative aging, Alzheimer’s disease, and Parkinson’s disease, and discusses multimodal integration, neuromodulation strategies, ethical considerations, and translational relevance for human neuroscience. Sections are organized around five linked goals: establishing EEG measurement validity in primates, summarizing aging and disease-associated EEG signatures, integrating EEG with imaging and invasive recordings, using stimulation to test causality, and translating biomarkers and interventions to human studies.

## 1 Introduction

The pursuit of understanding how brain function changes across aging and in neurodegenerative diseases has increasingly relied on model organisms capable of replicating human-like neural organization and network dynamics (Verdier et al., 2015). While rodent models have contributed substantial foundational knowledge, their neuroanatomical and cognitive dissimilarities limit the direct translation of findings to humans. Non-human primates (NHPs), by contrast, exhibit a cortical architecture and a repertoire of complex behaviors that more closely parallel human capabilities, thereby providing a highly relevant framework for mechanistic investigation (Verdier et al., 2015). In NHPs, the expanded and differentiated neocortex supports advanced sensory processing, executive control, and flexible learning, enabling task designs aligned with human paradigms and longitudinal designs that permit invasive validation not feasible in humans because of ethical and surgical constraints.

Electroencephalography (EEG) has emerged as a powerful, non-invasive technique for recording neuronal population activity from the scalp, offering millisecond-scale temporal resolution and enabling continuous monitoring of brain function across weeks, months, or even years in NHPs (Itoh et al., 2015). In human studies, EEG has consistently identified age-associated slowing of dominant oscillatory rhythms, alterations in network connectivity, and changes in event-related potentials. These neural markers have been proposed as candidate biomarkers for age-related cognitive decline and for tracking disease progression in conditions such as Alzheimer’s and Parkinson’s diseases (Meghdadi et al., 2021; Rodríguez-Serrano et al., 2024; Molcho et al., 2025). Across primate aging and disease models, candidate signatures include shifts toward lower-frequency activity, changes in long-range synchrony, and altered evoked response timing, which can be compared directly with human EEG phenotypes. In parallel, primate work enables causal tests by quantifying how stimulation protocols alter oscillatory power or phase locking and how these changes relate to behavioral performance or disease-relevant phenotypes.

Transferring these methodologies to NHP models allows for direct testing of mechanistic hypotheses in a brain that shares significant anatomical and functional similarities with humans. This enables controlled tests of mechanisms and interventions in a model close to humans before translation to clinical studies (Verdier et al., 2015). Species differences in skull and scalp musculature increase vulnerability to electromyographic contamination, and movement or restraint-related artifacts can further complicate signal quality (Nakamura et al., 2020).

While invasive approaches such as electrocorticography (ECoG) deliver superior spatial resolution and higher signal-to-noise ratios by bypassing the skull and scalp, they involve surgical procedures that introduce health risks and potential welfare concerns. Therefore, methodological choices often require balancing scientific objectives with ethical responsibility toward the animal’s well-being.

Importantly, the growing body of NHP research has begun to identify EEG signatures associated with normative aging as well as with Alzheimer’s disease and Parkinson’s disease models (Davin et al., 2022; Qiao et al., 2023; Ouchi et al., 2025). This has opened the possibility of comparing patterns across species to determine the degree of correspondence and divergence between primate models and human pathology. Beyond EEG alone, integration with other neuroimaging and neurophysiological modalities, such as magnetoencephalography (MEG), functional MRI (fMRI), and transcranial stimulation, provides a multimodal framework for identifying convergent biomarkers and testing targeted interventions (Zhongming et al., 2006; Engemann et al., 2020).

In this review, we present a comprehensive overview of EEG methodologies in NHPs, with particular emphasis on their application to studies of aging, Alzheimer’s disease, and Parkinson’s disease. We examine strategies for integrating EEG with other neurotechnologies and neuromodulation techniques, consider the ethical dimensions of such research, and discuss the translational implications of NHP EEG findings for human neuroscience. This review is organized around five linked goals: validating EEG measurement approaches in primates, summarizing EEG signatures of normal aging and neurodegenerative disease models, integrating EEG with imaging and invasive electrophysiology to improve interpretability, using neuromodulation with EEG readouts to test causality, and translating candidate biomarkers and interventions to human studies. Section 3 focuses on EEG acquisition and technical constraints; Section 4 summarizes age-related and disease-related EEG signatures in primate models; Section 5 discusses multimodal combinations that improve inference about generators and networks; Section 6 evaluates neuromodulation studies with neural readouts; and Sections 7 and 8 synthesize ethical considerations, translational implications, and prioritized future research directions. Because many reported aging and disease effects depend strongly on vigilance state, muscle contamination, and referencing choices, we first describe the method of literature search in Section 2 followed by acquisition decisions that shape what can be interpreted as a candidate biomarker in Section 3.

## 2 Methods

### 2.1 Study design

This article is a narrative review rather than a systematic review or meta-analysis. The goal was to synthesize study-level evidence and methodological considerations for EEG and EEG-linked neurotechnologies in non-human primates (NHPs), with emphasis on aging, Alzheimer’s disease models, and Parkinson’s disease models. Because the scope spans heterogeneous designs and outcome measures, we did not perform a formal risk-of-bias assessment or PRISMA-style study accounting; instead, we report a transparent search scope and explicit inclusion logic to reduce selection bias.

### 2.2 Literature search

Literature was identified through searches in PubMed, Web of Science, and Google Scholar. Searches were restricted to English-language records published between 2000 and January 2026. Search strings combined non-human primate terms (for example macaque, rhesus, marmoset, baboon, primate) with modality terms (EEG, ECoG, LFP, MEG, fMRI, fNIRS) and application terms (aging, Alzheimer, Parkinson, MPTP, amyloid, tau, biomarker, mismatch negativity, auditory steady-state response, TMS, tDCS, tACS, temporal interference, focused ultrasound). Backward and forward citation screening of key papers and relevant reviews was used to identify additional studies. The search process is reported with enough detail to support replication.

### 2.3 Eligibility criteria (NHP primary evidence)

Studies were eligible if they (i) included non-human primate data and (ii) reported EEG or closely related electrophysiology (scalp EEG, ECoG, and or depth LFP) in the context of aging, neurodegenerative disease models, multimodal integration, or neuromodulation with neural readouts. Studies were excluded if they did not include primate data, reported only behavior without neural recordings, or were not relevant to aging, Alzheimer’s disease, Parkinson’s disease, multimodal integration, or neuromodulation endpoints discussed here. Peer-reviewed journal articles were prioritized. Preprints were included only when they provided method-critical details not yet available in peer-reviewed form, and they were not used as the sole support for key conclusions.

### 2.4 Eligibility criteria (human-only translational evidence)

Human-only studies were included when they provided translational anchors that directly shaped interpretation or design of primate work in this review, such as (i) widely used clinical EEG biomarkers or paradigms that are explicitly mirrored in NHP studies (for example EEG slowing indices, MMN, ASSR), (ii) neuromodulation-EEG concepts and outcome measures used to motivate NHP parameter choices and readouts, or (iii) quantitative band definitions and reporting conventions needed for cross-species comparison. Human-only studies were excluded when they did not inform primate biomarker selection, primate experimental design, or cross-species interpretability (for example purely clinical prediction studies without a primate-mappable neural endpoint).

### 2.5 Screening and synthesis

Titles and abstracts were screened independently by two authors; disagreements were resolved by discussion, followed by full-text review of potentially eligible records. The evidence was synthesized qualitatively by organizing studies into acquisition constraints, aging and disease-associated EEG signatures, multimodal integration, and neuromodulation with neural readouts. When findings differed across reports, brain state, artifact burden, montage and referencing, and recording modality (scalp EEG versus invasive) were treated as primary sources of heterogeneity and described explicitly.

## 3 EEG recording in NHPs: methodologies and challenges

**FIGURE 1:**
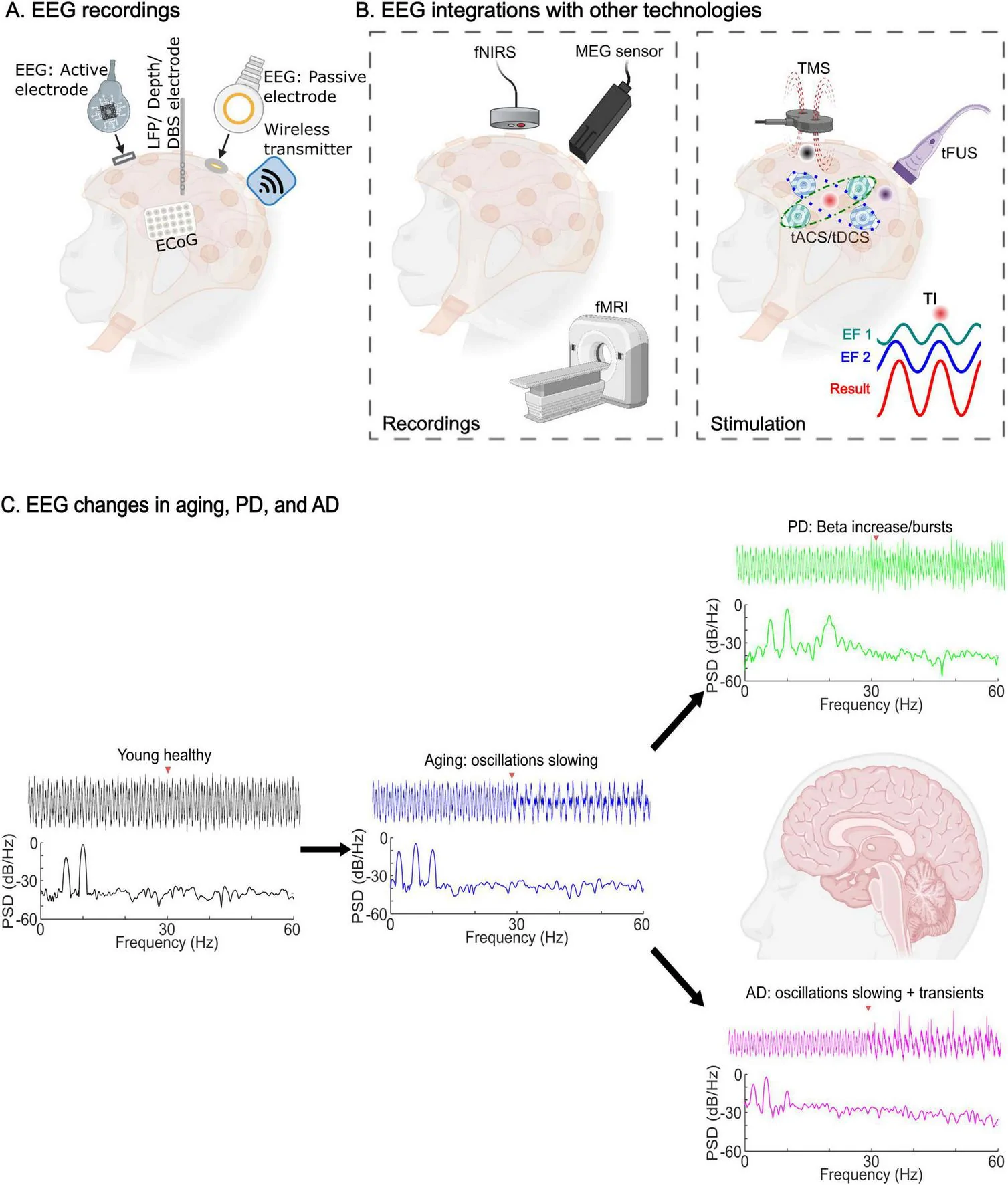
Non-human primate electroencephalography (NHP EEG) neurotechnology landscape and pathological signatures. **(A)** Methodology and Data Acquisition: Illustrates the choice between non-invasive scalp EEG and invasive approaches like electrocorticography (ECoG) and local field potentials (LFP). Non-invasive recordings utilize active electrodes to tolerate higher impedance or passive electrodes for high-fidelity baselines in low-motion settings. Wireless transmitters and headstages enable long-duration monitoring of ethological behaviors and sleep, while invasive probes provide “ground-truth” validation of deep-source activity. **(B)** Multimodal Integration and Causal Probing: (Left) EEG is paired with spatially resolved modalities including fMRI, MEG, and fNIRS to link millisecond-scale timing to network-level recruitment and vascular readouts. (Right) Integrated stimulation tools such as TMS, tDCS/tACS, Temporal Interference (TI), and tFUS enable causal testing by perturbing or entraining identified neural rhythms to observe behavioral consequences. **(C)** Pathological EEG Signatures: Summarizes the quantitative anchors of brain aging and neurodegeneration using simulated LFP data and their corresponding PSDs. Note the control LFP activity is prior to the red marker and the condition specific changes in LFP are shown on the right of the marker. Young Healthy: Displays a balanced power spectral density (PSD) with prominent alpha and gamma rhythms. Aging: Characterized by EEG slowing, visualized as a shift toward increased theta (4–8 Hz) and delta activity with a concomitant decrease in alpha power. Parkinson’s disease (PD): Marked by the emergence of pathological beta-band oscillations (13–30 Hz) and increased beta-burst dynamics in cortico-basal ganglia loops. Alzheimer’s disease (AD): Defined by severe oscillations slowing, increased low-frequency power, and network instability reflected in interictal-like transients and reduced 40 Hz Auditory Steady-State Responses (ASSR).

**FIGURE 2:**
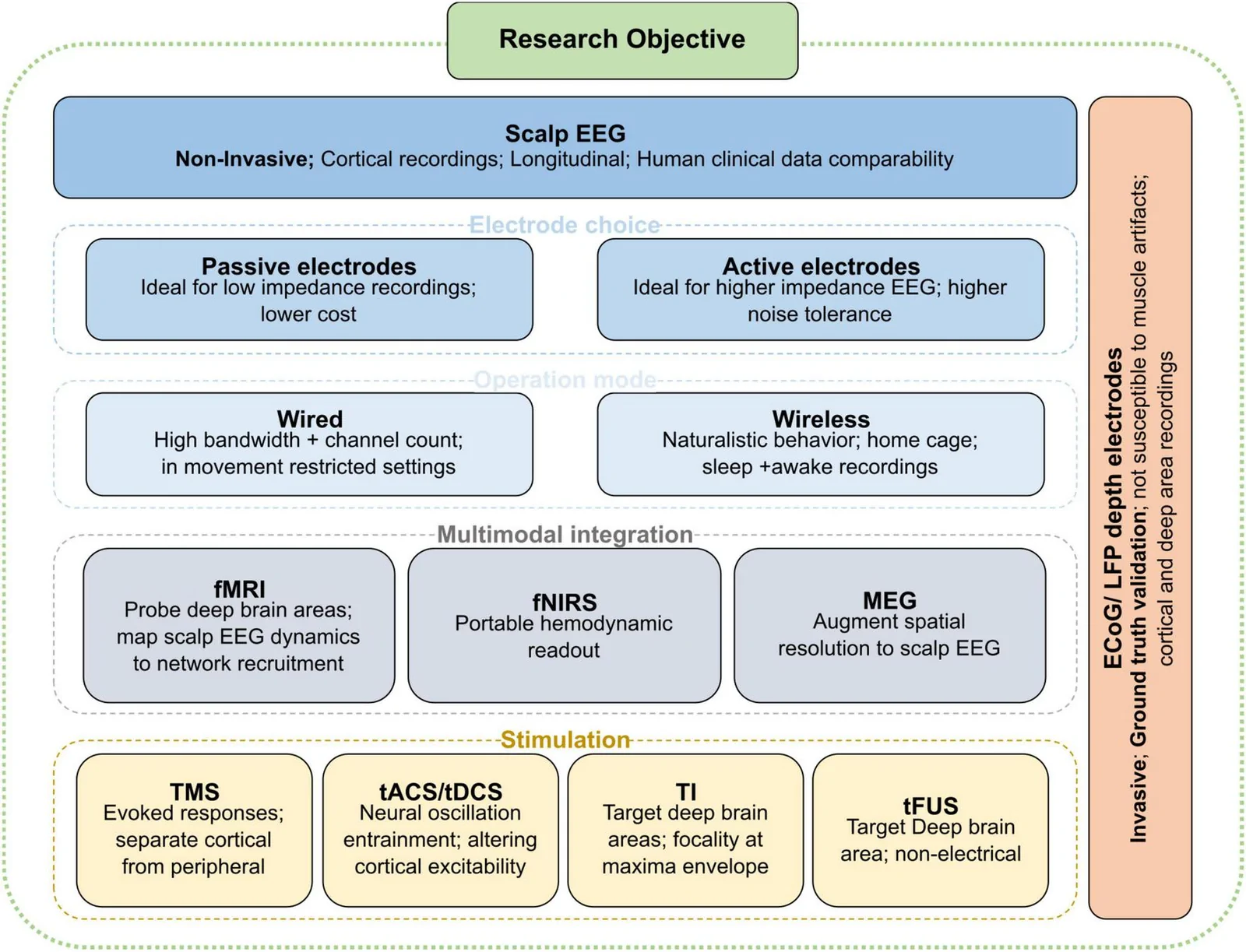
Strategic decision tree for NHP neurotechnology selection. This figure provides a logic-driven framework for selecting appropriate NHP neurotechnology based on the Research Objective. It condenses technical trade-offs into a practical tool for experimental design, helping to de-risk subsequent human clinical trials. Primary Choice: Selection of Scalp EEG for longitudinal studies and clinical comparability versus Invasive ECoG/LFP for ground-truth validation and deep area recordings (e.g., hippocampus or basal ganglia). Logistics and Operation: Defines the choice of Passive electrodes for low-impedance recordings or Active electrodes for higher noise tolerance. Wired systems are prioritized for high-bandwidth cognitive tasks, while Wireless systems are selected for naturalistic behavior and sleep monitoring. Multimodal and Stimulation: Guides the integration of imaging tools (e.g., fMRI to probe deep networks) and the application of specific neuromodulation strategies such as TMS for evoked responses or TI/tFUS for non-invasive deep-target engagement to test mechanistic hypotheses.

This section provides practical guidance for acquiring EEG in non-human primates and clarifies how acquisition choices constrain downstream inferences about aging and neurodegeneration. Primate EEG faces recurring challenges, including scalp morphology and muscle-related electromyographic contamination, variable electrode contact, and motion-related artifacts, which can bias spectral power, connectivity, and event-related measures if not addressed systematically. We therefore organize Section 3 around key decision points: non-invasive scalp EEG versus invasive ECoG and LFP for ground-truth validation, passive versus active electrodes (including impedance tolerance and susceptibility to environmental noise), and wired versus wireless systems (including movement constraints and recording duration limits). An overview of the main EEG acquisition and hardware choices is shown in Figure 1A. A practical decision tree for selecting recording modality, electrode technology, and logistics based on study objectives is provided in Figure 2. Throughout, we highlight implementation details that directly affect data quality, such as impedance targets, acclimation and training requirements, and artifact mitigation steps, to support reproducible comparisons across studies and species.

When designing a primate EEG study, researchers face a series of interdependent choices. For longitudinal biomarker studies in aging or neurodegeneration, non-invasive scalp EEG is preferred: it avoids surgical risks, permits repeated sampling over months or years, and yields data directly comparable to human EEG clinical recordings. For mechanistic studies where ground truth is essential (e.g., determining which scalp frequencies reflect specific deep sources), concurrent ECoG or depth LFP alongside scalp EEG is invaluable, though it requires surgical implantation and thus shorter time windows. For studies prioritizing animal welfare and natural behavior (e.g., sleep, social interactions, free locomotion), wireless systems are transformative, even if they trade some channel count or sampling rate for behavioral freedom. The following subsections detail each modality’s implementation specifics, allowing researchers to align their technical choices with their scientific questions. A summary of NHP EEG recording modalities and system trade-offs is provided in Table 1.

**TABLE 1:** Electroencephalography (EEG) recording modalities in non-human primates: scalp, invasive, and system trade-offs.

Category approach	Typical NHP use case	Main advantages	Key limitations/confounds to state	Key references
EEG acquisition: Scalp EEG (caps, primate 10–20 style)	Longitudinal aging and disease phenotyping and ERP paradigms with direct comparability to human clinical EEG.	Non-invasive and repeatable, with cross-species comparability.	EMG contamination, motion artifacts, and strong referencing and vigilance-state sensitivity.	Itoh et al., 2015; Li and Teichert, 2020; Nakamura et al., 2020.
EEG acquisition: High-density or stable-contact scalp solutions	Higher channel count and stable electrode contact in smaller primates (for example marmoset).	Enables improved topographic mapping and repeated acquisition in awake animals.	Contact stability and impedance drift across sessions.	Itoh et al., 2022.
EEG hardware: Active vs. passive electrodes	Active electrodes used when consistent low impedance is hard to maintain in primates.	Active electrodes tolerate higher impedance and reduce susceptibility to environmental noise.	Passive can be best only at very low impedance; active adds cost and has amplifier dynamics considerations.	Laszlo et al., 2014; Novembre et al., 2024.
Invasive validation: ECoG/μECoG	Ground-truth validation and higher SNR recordings during behavior; local re-referencing to reduce artifacts.	Higher amplitude and spatial resolution than scalp EEG and reduced cranial muscle artifacts.	Requires surgery and added welfare burden; typically justified for mechanistic validation rather than routine longitudinal monitoring.	Fukushima et al., 2015.
Invasive validation: Depth LFP	Deep generator access (hippocampus, basal ganglia) for mechanistic anchoring and linking scalp features to circuit activity.	Direct access to deep sources not reliably seen at scalp and frequency-resolved circuit biomarkers.	Surgical burden and dependence on targeting accuracy.	Gupta et al., 2025; Bimbi et al., 2018.
Recording logistics: Wired vs. wireless	Wired for maximal fidelity in seated tasks; wireless for sleep and ethological behavior with long recordings.	Wireless enables freer behavior and longer sampling; wired supports high bandwidth and channel count.	Wireless power and bandwidth constraints and telemetry risks; wired restrictions and cable-motion artifacts and stress without habituation.	Kaiju et al., 2025; Chestek et al., 2009; Fernandez-Leon et al., 2015.

Section 3 is organized as a set of acquisition decisions that directly determine which EEG features are interpretable as candidate aging or neurodegeneration biomarkers. For each decision, we summarize what the choice enables, the main failure modes (for example EMG contamination, state confounds, and montage or reference sensitivity), and a minimum reporting set to support cross-study comparison.

### 3.1 Non-invasive scalp EEG techniques in primates

Non-invasive scalp EEG is often preferred for longitudinal NHP studies because it avoids chronic cortical implantation while preserving direct comparability with human EEG phenotypes. In practice, primate scalp EEG requires species-specific cap or electrode-holder geometry, careful management of hair and impedance drift, and procedures that minimize head and jaw motion to limit cranial muscle contamination of spectral power and ERP measures. In alert rhesus macaques, Itoh et al. (2015) demonstrated feasible cortical auditory evoked potential (CAEP) recordings using a primate 10–20 style montage with up to 19 channels, including an EOG channel, while animals were seated in a monkey chair with partial head-motion restriction (Itoh et al., 2015). Here, “primate 10–20 equivalent” refers to an approximate mapping of the human International 10–20 layout onto the NHP head using homologous cranial landmarks and proportional spacing, recognizing that a single universally accepted NHP standard does not yet exist (Li and Teichert, 2020). Their CAEP components (mP1, mN1, mP2, mN2 and a sustained potential) showed consistent scalp topographies, supporting repeated ERP acquisition when ocular activity and motion are monitored.

In smaller primates, maintaining high channel counts with stable contact can be a limiting factor, and Itoh et al. (2022) addressed this in awake common marmosets using a “dip-in electrode” method. Specifically, gel-filled silicone tubes (4 mm internal diameter) were fixed to shaved scalp to mechanically isolate electrodes across channels, enabling 32-channel recordings with a minimum inter-electrode spacing of 6 mm and supporting topographic mapping of auditory evoked responses (Itoh et al., 2022). Complementing these montage and application strategies, Orczyk et al. (2021) provide an example from macaque face-ERP recordings showing that electrode configurations can be optimized to better sample targeted generators, including responses linked to superior temporal sulcus, while reducing temporalis EMG contamination. Finally, Szabó et al. (2005) offer a practical clinical-style scalp EEG reference in baboons, using shaved scalp with gold-cup electrodes fixed with collodion in a standard 10–20 montage (FP1/FP2/T7/T8/C3/C4/Cz/O1/O2/A1/A2), with impedance kept below 5 kΩ and additional EOG, EMG, and EKG channels for artifact and state monitoring (Szabó et al., 2005).

Scalp EEG is best suited for longitudinal sampling and direct cross-species alignment with clinical EEG, but interpretability is limited when candidate biomarkers depend on deep generators or when cranial muscle activity dominates the spectrum. Studies that aim to interpret frequency-specific changes should state how jaw and facial EMG were monitored or minimized, and should report how referencing choices affect the direction of spectral and connectivity results.

### 3.2 Active and passive EEG electrode technologies

Electrode design strongly influences signal quality, setup time, and feasibility in non-human primate EEG. Conventional passive electrodes, typically Ag/AgCl or gold-cup designs, generally require skin preparation and conductive paste or gel to achieve low impedance and stable contact. In controlled, low-motion settings this approach can yield excellent data quality, but it can be vulnerable to hair regrowth, movement, and cable-related pickup of environmental electrical noise, making careful shielding, grounding, and cable management important (Laszlo et al., 2014).

Active electrodes incorporate miniature preamplifiers within the electrode housing so that the signal is amplified at the point of contact before transmission through the lead wires. This reduces susceptibility to ambient electrical contamination and makes active systems more tolerant to higher skin-electrode impedance, which can be advantageous when consistently low impedances are difficult to maintain in primates (Laszlo et al., 2014). In a direct within-system comparison, passive electrodes produced the cleanest ERP baselines only under very low impedance conditions (approximately 2 kΩ), whereas at higher impedances active electrodes outperformed passive electrodes and were less sensitive to impedance increases overall (Laszlo et al., 2014). Practical tradeoffs include higher cost and the possibility of reduced accuracy during rapid voltage changes due to amplifier dynamics, which may be relevant for fast ERP components and EOG-rich epochs (Laszlo et al., 2014). Active electrodes have been used successfully in awake rhesus macaques, for example using a 29-electrode BioSemi Active-2 system mounted on custom-fitted caps (Novembre et al., 2024), and they can also support lower-stress acquisition approaches when combined with protocols that prioritize motion reduction and animal welfare (Nakamura et al., 2020; Piette et al., 2025).

For longitudinal primate cohorts where impedance drift and motion are expected, active electrodes can reduce sensitivity to cable and environmental noise at higher impedances. For low-motion, well-controlled settings where very low impedance can be maintained reliably, passive electrodes can yield stable ERP baselines. Regardless of electrode type, studies should report impedance distributions over sessions, not only a single threshold, because aging and repeated setup can shift contact quality over time.

### 3.3 Invasive electrocorticography (ECoG) and LFP recording

Electrocorticography (ECoG) provides higher-amplitude signals and improved spatial resolution compared with scalp EEG because recordings are obtained directly from the cortical surface, largely bypassing skull/scalp attenuation and reducing sensitivity to cranial muscle artifacts. In non-human primates (NHPs), flexible ECoG and μECoG arrays can conform to gyral curvature and support higher-density sampling across targeted cortical territories, enabling detailed mapping of sensory-evoked responses, oscillatory dynamics, and mesoscale connectivity patterns. For example, Fukushima et al. (2015) describe a chronic macaque ECoG array approach designed to record simultaneously from broad cortical extents, including medial and lateral surfaces and intrasulcal cortex (e.g., supratemporal plane), which is particularly useful for network-level questions that extend beyond a single gyrus.

A practical advantage of ECoG in awake, behaving primates is improved artifact handling relative to scalp EEG, especially when recordings can be locally re-referenced. In the Fukushima et al. (2015) implementation, bipolar recordings from adjacent electrode pairs reduced chewing (mastication) artifacts that were evident in monopolar recordings, illustrating how montage choices at the intracranial level can materially improve data quality during natural behaviors. These considerations are relevant for aging and neurodegeneration studies that often prioritize longitudinal sampling, task engagement, and/or ethologically richer settings where cranial muscle contamination can otherwise bias spectral and connectivity measures.

Beyond the cortical surface, depth electrodes and chronic intracortical/laminar probes enable local field potential (LFP) measurements within deep cortical and subcortical structures, including hippocampal and basal ganglia circuits that are difficult to access with scalp EEG alone (Gupta et al., 2023). This ability to sample deep generators is increasingly important for mechanistic studies of memory circuits and for neurodegeneration models where subcortical–cortical interactions are central to pathophysiology. Because deep recordings depend strongly on targeting accuracy in macaques, high-precision implantation workflows are a key enabler of chronic LFP studies; for instance, Gupta et al. (2025) describe a multi-camera neuronavigation framework combined with MRI/CT guidance for chronic implantation of depth electrodes targeting hippocampus and entorhinal cortex in macaques, reporting submillimetric-to-millimetric targeting accuracy and detailing a replicable surgical workflow.

Finally, simultaneous scalp and invasive recordings (ECoG and/or depth LFP) provide a direct bridge between “ground truth” neural dynamics and what is measurable at the scalp, enabling explicit quantification of frequency-dependent attenuation and distortion introduced by intervening tissues. Such concurrent recordings can improve interpretability of candidate EEG biomarkers by clarifying which intracranial rhythms plausibly contribute to scalp-band power or phase metrics under a given behavioral state. As a concrete demonstration of invasive–non-invasive coupling in macaques, Bimbi et al. (2018) combined scalp EEG with intracortical signals in ventral premotor cortex during action execution/observation and showed that increases in local high-gamma LFP power correlate with (and can precede) central scalp beta desynchronization.

Despite their scientific value, ECoG/LFP approaches require surgery (e.g., craniotomy and/or chronic chambers), impose higher operational complexity, and carry additional welfare considerations; therefore, they are typically best justified for targeted mechanistic validation or for developing/anchoring non-invasive biomarkers rather than for routine longitudinal monitoring. Invasive recordings strengthen biomarker claims by testing whether a scalp feature reflects a genuine circuit rhythm versus a state or muscle artifact, and by quantifying how frequency-dependent attenuation and re-referencing choices shape what is visible at the scalp.

### 3.4 Wired vs. wireless systems: trade-offs and use cases

Traditionally, primate EEG and ECoG experiments have relied on wired (tethered) recording systems, in which the animal is connected via a cable to external amplifiers and acquisition hardware. Wired setups can support high channel counts and wide bandwidth, but they restrict the animal’s movement and can introduce artifacts from cable motion and mechanical strain at the connector-headstage interface. To reduce entanglement risk and stabilize recordings, tethering is often paired with chair restraint, which limits natural behaviors and can increase stress if habituation and training are insufficient (Chestek et al., 2009; Fernandez-Leon et al., 2015; Yan T. et al., 2023; Kaiju et al., 2025).

Wireless EEG/ECoG systems address these constraints by shifting amplification and/or digitization onto a headstage and transmitting data to a receiver, enabling freer movement and, in some cases, recording in the home cage. This facilitates more ethologically relevant experiments (e.g., social interactions and free exploration) and can improve animal welfare and data quantity by allowing longer and more frequent recordings (Kaiju et al., 2025). The main trade-offs are power and bandwidth limitations (battery-constrained duration), potential reductions in channel count and/or sampling rate compared with wired systems, and engineering challenges in maintaining low-noise acquisition with robust synchronization and minimal data loss during telemetry (Kaiju et al., 2025).

For laboratory cognitive tasks where the monkey is trained to sit calmly and the priority is maximal signal fidelity (e.g., high bandwidth, stable referencing, and minimal motion artifact), wired or lightly tethered configurations are often the most appropriate choice. For studies centered on sleep, long-duration monitoring, or more ethologically relevant behavior, contexts where brain state sampling and ecological validity are priorities, wireless systems are often preferable despite current technical constraints, because they provide access to behavioral states that are otherwise difficult to measure repeatedly and at scale (Kaiju et al., 2025).

For aging and neurodegeneration cohorts, the main scientific advantage of wireless systems is expanded access to vigilance states and naturalistic behavior, which can reduce sampling bias toward chair-restrained quiet wake. Wired systems remain preferable when the primary endpoint requires maximal bandwidth, stable referencing, and controlled task epochs, but authors should state explicitly which states were sampled and which were not, because this choice can shift spectral and connectivity outcomes

Acquisition choices (scalp vs. invasive validation, passive vs. active electrodes, wired vs. wireless) shape the dominant artifact sources and behavioral states that can be sampled, and therefore constrain which spectral, connectivity, and ERP metrics are interpretable. These choices most directly affect the reliability of spectral power, coherence, and ERP measures that are commonly used as aging and disease readouts in Section 4. This matters directly for aging and neurodegeneration, where many reported EEG effects depend on vigilance state (wake vs. drowsiness vs. sleep), movement/EMG burden, and the feasibility of repeated measurements over months to years. With these technical constraints made explicit, Section 4 summarizes the EEG signatures reported in NHP models of normative aging and neurodegenerative disease and highlights how recording context and state sampling shape the direction and reliability of those signatures.

Taken together, these acquisition decisions determine which EEG features can be interpreted as candidate aging or disease biomarkers versus artifacts of state, EMG, or referencing. Across studies, the main sources of cross-report disagreement are vigilance-state sampling, cranial muscle contamination, and montage-dependent shifts in spectral and connectivity measures. As a result, claims about biomarker specificity should be weighted more heavily when studies report explicit state monitoring, EMG control, and where possible concurrent invasive validation. With these constraints in view, the next section focuses on which spectral, ERP, and connectivity signatures show consistent age or disease associations in primate models, and where evidence remains limited by cohort size, model heterogeneity, or recording context.

## 4 EEG signatures of aging and neurodegeneration in NHPs

Aging brains exhibit characteristic shifts in oscillatory activity, spectral power distribution, network coherence, and event-related potential timing. By recording EEG in aged versus young primates, researchers can identify electrophysiological hallmarks of normal cognitive aging and distinguish them from early pathological changes (Barbosa et al., 2020; Upright and Baxter, 2021; Gupta et al., 2023). This section synthesizes findings across three domains: normative aging, Alzheimer’s disease, and Parkinson’s disease models in NHPs. A central goal is to evaluate how candidate EEG biomarkers identified in primates compare with human phenotypes and whether specific oscillatory or connectivity abnormalities can predict behavioral decline (Figure 1C). Moreover, in NHP models of neurodegenerative diseases like Alzheimer’s and Parkinson’s, EEG is used to uncover disease-specific neural signatures (for example, characteristic slowing of dominant rhythms, increased low-frequency power, enhanced theta-gamma coupling, or the emergence of epileptiform activity) that could translate to early biomarkers and intervention targets in humans (Yu T. et al., 2021; Davin et al., 2022). The empirical evidence reviewed here demonstrates a strong correspondence between primate and human EEG patterns in aging and disease, supporting the translational value of NHP models for biomarker discovery and therapeutic development. Recent studies have also examined how different recording modalities (scalp EEG versus invasive ECoG and LFP) and behavioral states (wakefulness, sleep, and anesthesia) influence the detectability and reliability of these markers, issues that are critical for interpreting cross-species comparisons.

### 4.1 Normal aging and cognitive decline: EEG insights

Even in the absence of overt disease, normal aging leads to subtle cognitive decline; for example, older primates show slower learning, reduced working memory, and changes in executive function (Upright and Baxter, 2021; Frye et al., 2022). In humans, healthy aging often entails a slowing of EEG rhythms, including reduced alpha power and increased theta/delta activity, along with reduced EEG complexity (Jelic et al., 1996; Babiloni et al., 2006; Burke and Barnes, 2010; Lacreuse et al., 2020; Yu T. et al., 2021; Jia et al., 2023; Azami et al., 2025; Xin et al., 2025). Studies in rhesus macaques and marmosets suggest parallel trends. In macaques, aged animals (commonly 20+ years old), tend to have diminished high-frequency oscillations during cognitive tasks and weaker event-related potentials, consistent with slower cortical processing (Peters et al., 1996; Moss et al., 1999; Stonebarger et al., 2021). For instance, aged macaques had longer latencies and smaller amplitudes of visually evoked potentials compared to young adults, consistent with delayed neural conduction (Ibáñez-Contreras et al., 2018). Additionally, spontaneous EEG from older monkeys can exhibit an increased ratio of low-frequency theta band (4–8 Hz) to alpha-band activity, analogous to the EEG slowing seen in human seniors (Jabès et al., 2021; Merkin et al., 2021). Notably, aged NHPs also spontaneously develop some neuropathological changes including amyloid plaques and hyperphosphorylated tau in the cortex, which may contribute to EEG changes (Oikawa et al., 2010); comparative neuropathology reviews further suggest that beta-amyloid deposition (including cerebral amyloid angiopathy) is frequent in aged NHPs, whereas tau pathology is generally mild or moderate (Isidro, 2024). Age-related cognitive decline and Alzheimer-related pathophysiology have been linked to neural hyperexcitability and altered network synchronization, raising the possibility that some age-associated EEG changes reflect network destabilization rather than passive slowing alone (Haberman et al., 2017; Yu T. et al., 2021). Because aging in rhesus macaques is also associated with changes in sleep quantity and quality and reduced stability of circadian rhythms, EEG aging markers should be interpreted in relation to vigilance state and acquisition context, especially when comparing across studies or cohorts (Zhdanova et al., 2011; Nakamura et al., 2020).

### 4.2 EEG biomarkers in primate models of Alzheimer’s disease

Alzheimer’s disease (AD) relevant pathology occurs in aged NHPs, and additional NHP AD models use induced approaches (for example, administration of Aβ-related materials such as brain homogenates) or genetically modified lines, creating a spectrum of model types with different strengths for biomarker development and mechanistic testing (Horváth et al., 2016; Palop and Mucke, 2016; Wong et al., 2022). Comparative primate neuropathology further suggests that Aβ deposition (including cerebral amyloid angiopathy) is frequent in aged NHPs, whereas tau pathology is often milder than in human AD, which is important when interpreting EEG phenotypes as “AD-like” versus “aging-like” (Isidro, 2024). Within this context, EEG is increasingly used in NHP models to identify translational biomarkers that can be compared directly with human EEG signatures and, where possible, anchored to invasive or molecular readouts.

Electroencephalography slowing is one candidate biomarker that parallels human findings, where AD is often associated with reduced alpha (8–12 Hz) and increased theta (4–7 Hz) activity (Palop and Mucke, 2016; Wong et al., 2022). In a primate study using inoculation with brain extracts from AD patients to induce pathology, scalp EEG spectral power shifted, with lower delta power and higher theta power relative to controls, providing a concrete example of a frequency-domain EEG phenotype emerging following AD-relevant exposure (Wong et al., 2022). These observations support the use of longitudinal spectral metrics (for example, alpha-to-theta balance and related slowing indices) as candidate readouts for disease progression and for intervention testing in NHP cohorts, while keeping in mind that scalp EEG spectra are sensitive to vigilance state and acquisition context.

A second candidate domain is network instability and hyperexcitability. Convergent evidence from AD research links early disease processes to aberrant excitability and epileptiform activity, which can precede major cognitive decline and may relate to altered theta and gamma dynamics (Horváth et al., 2016; Palop and Mucke, 2016; Wong et al., 2022). In NHP AD paradigms, prolonged recordings and standardized detection of interictal-like events represent a practical next step to determine when and where epileptiform transients emerge, and whether they covary with behavioral decline or molecular markers (Horváth et al., 2016; Palop and Mucke, 2016; Wong et al., 2022). This is also a setting where concurrent invasive recordings can be particularly informative for clarifying which deep or mesial temporal events can be detected reliably at the scalp.

Event-related EEG measures provide a complementary class of biomarkers that are less dependent on resting-state state fluctuations and can be aligned with human paradigms. Auditory mismatch negativity (MMN) and auditory steady-state responses (ASSR) have been proposed as functional biomarkers of cognitive impairment and circuit dysfunction, and early NHP work suggests that these measures can be acquired with sufficient reliability to support cross-species comparison (Tada et al., 2020; Nakamura et al., 2022). In marmoset work with amyloid-relevant pathology, declines in MMN amplitude and reduced gamma-band ASSR coherence have been reported, consistent with impaired sensory prediction and reduced gamma synchrony that are also discussed in human prodromal and clinical AD contexts (Tada et al., 2020). These paradigms are attractive because they can be repeated longitudinally and paired with pharmacological or stimulation interventions to test whether physiological normalization accompanies behavioral improvement.

A limitation across current NHP AD EEG studies is heterogeneity in model type, recording context (awake versus anesthetized), montage and referencing, and the duration of longitudinal sampling, which complicates direct comparison across cohorts and laboratories. Future work would benefit from aligning EEG endpoints to model stage using convergent measures (for example, PET, CSF biomarkers, or post-mortem confirmation when available), and from adopting harmonized preprocessing pipelines and state monitoring so that spectral and ERP biomarkers can be compared quantitatively across studies and species (Palop and Mucke, 2016; Wong et al., 2022).

### 4.3 EEG biomarkers in primate models of Parkinson’s disease

Parkinson’s disease (PD) research has long benefited from NHP models, particularly the MPTP-treated monkey, which replicates dopaminergic neuron loss and motor symptoms (AlShimemeri et al., 2021). EEG-based markers of PD in humans include exaggerated beta oscillations (∼13–30 Hz) in cortico-basal ganglia circuits and abnormalities in brain rhythm coupling during movement (Raz et al., 2000). Because these rhythms arise from recurrent interactions between cortex and basal ganglia (BG) nuclei, concurrent BG local field potential (LFP) recordings in NHPs provide an important bridge between circuit-level pathology and what is measurable on the scalp. In particular, BG LFPs can quantify beta-band synchronization within the basal ganglia network and help determine whether scalp beta changes reflect genuine network oscillations versus state- or muscle-related effects.

Monkeys with MPTP lesions exhibit analogous beta abnormalities. In one study with progressive MPTP administration in macaques, as the animals became parkinsonian, their awake EEG developed a prominent peak in the low-beta range (∼13 Hz) that was absent in the healthy state (Yu Y. et al., 2021; Davin et al., 2022). This spectral peak provides a within-subject example of a candidate EEG marker that emerges with disease expression under awake recording conditions. Complementing scalp measures, implanted-recording studies in parkinsonian NHPs demonstrate that LFPs can be acquired chronically from deep targets using clinical-style hardware, supporting longitudinal tracking of pathological oscillations and responses to therapy (Connolly et al., 2015). Such LFP recordings are well suited for testing whether scalp beta features (peak power, coherence, or burst-like events) co-vary with BG beta dynamics across disease progression and during dopaminergic or stimulation-based interventions.

This pathological beta oscillation is significant because it correlates with motor rigidity and bradykinesia; indeed, treatments that alleviate motor signs (like L-DOPA or deep brain stimulation) tend to suppress beta rhythms in both humans and monkeys. Additionally, PD model monkeys show disrupted modulation of sensorimotor rhythms. In normal conditions, cortical beta power transiently desynchronizes during movement initiation, a phenomenon called event-related desynchronization (ERD). In MPTP monkeys, EEG and ECoG recordings have revealed a reduction in such ERD, indicating rigidly synchronized networks that fail to flexibly engage and disengage during movements (Escola et al., 2003; Deffains and Bergman, 2019). Interpreting reduced ERD can be strengthened by parallel BG LFP measures, since persistent beta synchrony in BG loops can be expressed as diminished flexibility in cortical beta suppression during movement initiation.

Other EEG changes in parkinsonian primates include increased coherence between cortical areas at lower frequencies (for example excessive delta coherence), possibly reflecting compensatory coupling when dopaminergic drive is lost, and alterations in sleep EEG architecture since PD often disrupts sleep-wake cycles (Davin et al., 2022). Notably, longitudinal EEG tracking in MPTP monkeys has identified early changes even before overt motor symptoms; during the prodromal phase, animals showed subtle increases in synchronized beta and gamma activity during quiet waking, suggesting emerging network dysfunction (Fox and Brotchie, 2010; Little and Brown, 2014; Connolly et al., 2015). These findings illustrate how EEG in NHP PD models can reveal both consequences of dopamine depletion on cortical dynamics and candidate prodromal markers, while invasive BG LFP can provide mechanistic anchoring and validation of the oscillatory features inferred from the scalp. In the future, combining EEG with dopamine-level measurements or pharmacological challenges in monkeys could further dissect the circuitry changes underlying PD and guide development of EEG-based markers to monitor disease progression or treatment efficacy in patients.

Across aging and neurodegeneration models, several EEG signatures recur, but their direction and apparent specificity often depend on model stage, recording state, and whether measures are derived from scalp EEG versus invasive recordings. In particular, slowing-related metrics and network synchrony measures can reflect genuine circuit change, but they can also shift with drowsiness, medication, or increased artifact burden in aged animals. Therefore, evidence is strongest when EEG changes are linked to behavioral decline, neuropathology, or invasive circuit readouts within the same animals and across time. This motivates multimodal integration, where EEG is paired with imaging or invasive recordings to localize generators and separate neural mechanisms from state-dependent or measurement-dependent effects.

## 5 Multimodal integration of EEG with other neurotechnologies

While EEG alone is informative, combining it with complementary modalities improves inference about neural generators, network interactions, and disease mechanisms in primate models of aging and neurodegeneration. Scalp EEG provides millisecond timing but has limited spatial specificity and reduced sensitivity to deep sources, and many candidate biomarkers depend on vigilance state and artifacts that can be difficult to disambiguate using EEG alone. Multimodal designs (Figure 1B, left) address these constraints by linking EEG features to spatially resolved measures (MEG and fMRI), hemodynamic readouts (fNIRS), and invasive electrophysiology (ECoG and depth LFP) that can validate whether a scalp signature reflects a specific circuit mechanism. Representative multimodal EEG combinations in NHPs, with use cases and interpretive constraints, are summarized in Table 2.

**TABLE 2:** Multimodal EEG integration in non-human primates: use cases, advantages, limitations, and representative studies.

Approach	Typical NHP use case	Main advantages	Key limitations/confounds to state	Key references
EEG + MEG	Use MEG to localize frequency-domain effects and cross-validate oscillatory signatures beyond scalp EEG alone.	Adds complementary spatial sensitivity and regional localization compared to scalp EEG.	Inverse problem uncertainty; anesthesia and head-motion constraints can shape feasibility and interpretation.	Wilson et al., 2009; Zumer et al., 2010; Rowland et al., 2017; Sandhaeger et al., 2019.
EEG + fMRI	Link EEG timing to spatially resolved hemodynamics and test whether candidate EEG signatures map onto network recruitment or connectivity changes.	Supports translational bridging, especially when aligned with invasive electrophysiology-fMRI coupling results in NHPs.	Large MRI-induced EEG artifacts; interpretation requires explicit control of vigilance state, head motion, and physiological noise.	Schmid et al., 2006; Schölvinck et al., 2010; Hargreaves et al., 2012; Hutchison et al., 2015.
EEG + fNIRS	Portable hemodynamic readout to complement EEG, with NHP demonstrations during voluntary movement without head fixation and DOT-based reconstruction for improved anatomical interpretability.	Adds local vascular readout and can support repeated within-subject monitoring in more naturalistic settings than MRI.	Direct simultaneous EEG-fNIRS demonstrations in NHP remain limited versus EEG-fMRI or invasive validation; both modalities are motion and superficial-physiology sensitive.	Yamada et al., 2018; Hayashi et al., 2022; Wakita et al., 2010; Blanco et al., 2024; Aghajani et al., 2017.

In practice, integration is most useful when it is organized around a defined question, for example whether an apparent change in band power reflects altered cortical generators, altered coupling to subcortical loops, or altered neurovascular responses across aging or disease stage. However, multimodal recordings introduce additional technical and interpretive challenges, including cross-modal artifacts, electrode and sensor co-registration, synchronization accuracy, and the need to control behavioral and arousal state across sessions. The following subsections summarize established multimodal pairings in NHPs, focusing on what each combination adds beyond EEG alone and what types of biomarkers and mechanistic tests they enable in aging and neurodegeneration studies.

### 5.1 EEG and magnetoencephalography (MEG) in NHPs

Magnetoencephalography (MEG) measures the magnetic fields produced by neuronal currents and provides a complementary non-invasive readout to EEG. In macaques, MEG feasibility has been established under controlled conditions using human whole-head systems (Wilson et al., 2009; Sandhaeger et al., 2019). Zumer et al. (2010) recorded MEG in anesthetized macaques during tactile and auditory stimulation and showed that source reconstruction could differentiate primary somatosensory versus auditory cortex and could separate digit versus lip representations within somatosensory cortex. They also emphasize that MEG source localization carries non-trivial inverse uncertainty, citing phantom-based spatial uncertainty of about 3 mm (and potentially larger depending on modeling and assumptions), and report that auditory evoked fields were harder to detect than tactile responses in some animals (clear auditory responses in 5/13 blocks), consistent with generator geometry and signal-to-noise constraints in the temporal lobe (Zumer et al., 2010).

Complementing this work, Wilson et al. (2009) mapped somatosensory responses in rhesus monkeys with whole-head MEG and reported a marked latency difference compared with human reports, further supporting the feasibility of translating standard MEG evoked-field paradigms to NHPs.

Magnetoencephalography has also been used for resting-state spectral phenotyping in NHP models. In vervet monkeys, Rowland et al. (2017) performed whole-head resting-state MEG under propofol anesthesia (used to limit head motion) after long-term ethanol self-administration and reported localized power differences, including reduced power in anterior cingulate cortex, hippocampus, and amygdala, and increased power in right medial orbital and parietal regions. This study is useful for interpretation because it explicitly notes anesthesia as a confound, while also illustrating a practical advantage of MEG over scalp EEG: frequency-domain effects can be localized to specific regions rather than pooled across heterogeneous generators (Rowland et al., 2017).

For multimodal primate designs, EEG-MEG pairing can be used as a cross-validation step for oscillatory signatures and to prioritize candidate cortical generators for subsequent EEG-fMRI or invasive validation in later subsections (Wilson et al., 2009; Zumer et al., 2010).

### 5.2 Concurrent EEG and functional MRI (fMRI) in NHPs

Simultaneous EEG-fMRI links millisecond-scale electrophysiology to spatially-resolved hemodynamic changes but is technically challenging because MRI gradients and radiofrequency pulses induce large artifacts in EEG leads and electrodes. In macaques, Schmid et al. (2006) demonstrated feasibility of scalp EEG recorded during fMRI at 4.7 T in an awake behaving monkey and validated gradient-artifact correction in phantom recordings, reporting recovery of 91% of the amplitude of a 10 μV, 10 Hz phantom signal without phase distortion and reliable identification of visual evoked potentials during concurrent acquisition.

For primate aging and neurodegeneration models, EEG-fMRI can test whether candidate EEG signatures (for example slowing indices, burst statistics, or task-evoked dynamics) map onto changes in network recruitment, functional connectivity, or neurovascular coupling, but interpretation requires explicit control of vigilance state, head motion, and physiological noise because these factors can covary with both EEG spectra and BOLD fluctuations. A practical way to strengthen interpretation is to align EEG-fMRI outcomes with invasive electrophysiology-fMRI coupling results in NHPs, since implanted electrodes are less susceptible to MRI-related artifacts than scalp leads (Schmid et al., 2006). Task-based primate electrophysiology provides an additional anchor for interpreting hemodynamic signals. Hargreaves et al. (2012) analyzed macaque medial temporal lobe LFP during associative learning and compared it to human fMRI collected using the same task structure and parallel analyses, reporting conserved learning-related signals across hippocampus and entorhinal cortex and analyzing monkey LFP in beta (10–25 Hz) and gamma (30–100 Hz) ranges.

In awake macaques at rest, Schölvinck et al. (2010) combined intracranial LFP recordings with fMRI and showed that spontaneous global fMRI fluctuations correlate most consistently with upper gamma-band LFP power (40–80 Hz), while the strength of coupling depends on behavioral state (for example eyes open versus closed). Under anesthesia, Hutchison et al. (2015) recorded simultaneous intracortical LFPs from prefrontal cortex and 7 T fMRI in macaques and showed that band-limited power envelopes exhibit frequency-dependent relationships with spontaneous BOLD activity across connected regions, emphasizing that brain state can alter both spectral structure and LFP–BOLD coupling. Together, these studies motivate EEG-fMRI in NHP disease models as a translational bridge: scalp EEG supports longitudinal sampling, while invasive LFP-fMRI helps constrain which frequency-resolved features are most likely to reflect underlying population activity versus state or artifact (Schmid et al., 2006; Schölvinck et al., 2010; Hargreaves et al., 2012; Hutchison et al., 2015).

### 5.3 EEG and functional near-infrared spectroscopy (fNIRS) in NHPs

Functional near-infrared spectroscopy (fNIRS) is a portable optical method that tracks cortical hemodynamic changes through oxyhemoglobin and deoxyhemoglobin dynamics and can complement EEG by adding a local vascular readout (Blanco et al., 2024). While simultaneous EEG-fNIRS is widely used in humans for linking fast electrophysiological dynamics to slower hemodynamic responses, direct demonstrations in non-human primates remain relatively limited compared with EEG-fMRI or invasive validation approaches. In humans, hybrid EEG-fNIRS has been used in applied cognitive settings such as quantifying mental workload, illustrating that paired electrophysiological and hemodynamic readouts can be informative in paradigms where both fast dynamics and slower vascular responses change with task demands (Aghajani et al., 2017). A pragmatic view is therefore that NHP work currently relies more heavily on (i) establishing robust fNIRS acquisition during ethologically relevant behavior and (ii) translating human EEG-fNIRS analysis concepts cautiously, with explicit attention to motion and systemic physiology.

In macaques, Yamada et al. (2018) demonstrated fNIRS acquisition during voluntary movements without head fixation using a 27-channel configuration with 7.5 mm optode spacing and reported lateralized motor-cortex hemodynamic responses during repeated left versus right hand pellet retrieval. This provides a practical NHP use case for aging and disease cohorts: repeated within-subject monitoring of cortical activation patterns under relatively natural behavior constraints (Yamada et al., 2018).

Beyond channel-wise topography, diffuse optical tomography (DOT) can improve anatomical interpretability by reconstructing 3D hemodynamic changes from fNIRS measurements. Hayashi et al. (2022) applied DOT to macaque fNIRS recorded during left or right hand food retrieval under head-free conditions and reported contralateral increases in HbO and decreases in HbR near motor-related regions, supporting a tractable analysis workflow for multi-session monitoring.

Earlier feasibility work also supports the practicality of optical readouts in behaving macaques across task domains, including visually driven cognitive processing recorded without head fixation (Wakita et al., 2010). In principle, EEG-fNIRS pairing in NHPs can link fast oscillatory or event-related EEG dynamics to hemodynamic responses over the same cortical territory, but both modalities are sensitive to motion and superficial physiological contributions, so primate studies should report state control and artifact handling explicitly, especially for longitudinal aging or neurodegeneration designs.

Multimodal studies can substantially increase interpretability of EEG biomarkers, but they introduce their own constraints, including cross-modal artifacts, co-registration error, and differences in behavioral state across sessions. For many applications, the strongest inference comes from designs that explicitly test whether the same EEG feature maps to a consistent generator or network change across modalities and conditions. Remaining uncertainty is highest when studies lack invasive anchoring or when state differs across recordings. These limitations set up the rationale for the next section on neuromodulation, where stimulation combined with EEG or invasive recordings can be used to test causality by perturbing candidate circuits and quantifying target engagement.

## 6 EEG and neuromodulation in NHPs

Section 6 focuses on neuromodulation studies in non-human primates (NHPs) where EEG, ECoG, and or local field potentials serve as quantitative neural readouts of stimulation effects. The core goal is to link stimulation parameters to specific, testable changes in network dynamics that are relevant for aging and neurodegeneration, such as shifts in band-limited power, phase locking and coupling, evoked potential amplitudes and latencies, and changes in functional connectivity estimates. Across modalities, interpretation benefits when recordings are paired with explicit control of brain state (wake, sleep, anesthesia), careful handling of stimulation-related artifacts, and within-subject control conditions such as sham or parameter-matched comparisons. Practical use cases and key reporting confounds for stimulation combined with EEG or invasive electrophysiology are summarized in Table 3.

**TABLE 3:** Stimulation plus EEG in non-human primates: practical use cases and reporting confounds.

Approach	Typical NHP use case	Main advantages	Key limitations/confounds to state	Key references
TMS with EEG/ECoG/LFP	Perturbation mapping (evoked responses and propagation) and aftereffects, with invasive recordings helping separate cortical from peripheral components.	Causal perturbation with direct readouts of reactivity and propagation.	Coil positioning, artifact control, and strong state dependence of evoked responses.	Romero et al., 2019; Perera et al., 2023; Pigarev et al., 2008; Ohnishi et al., 2004; Baker et al., 1995.
tDCS/tACS	Link montage and dose to circuit-level effects using concurrent invasive recordings and EEG endpoints (including rhythm entrainment).	Frequency-specific intervention (tACS) with primate physiology supporting translational parameter selection.	During-stimulation artifacts (especially tACS), montage and field-distribution uncertainty, and state dependence.	Fehring et al., 2024; Krause et al., 2019; Johnson et al., 2020; Vieira et al., 2025; Hoffner et al., 2024.
Temporal interference (TI)	Deep-target ambition requiring explicit on-target vs. off-target validation with multi-site recordings and careful separation of carrier artifact vs. envelope-frequency physiology.	NHP work can define target-engagement criteria and quantify spike timing or phase-locking effects at the envelope frequency.	Mechanism debated; envelope maxima can occur in cortex as well as at depth; stimulation artifacts strongest at carrier frequencies.	Liu et al., 2024; Vieira et al., 2024; Mirzakhalili et al., 2020; Botzanowski et al., 2023; Yatsuda et al., 2024.
tFUS	Deep and focal modulation candidate; EEG suggested as a scalable readout but NHP tFUS-EEG evidence is less developed than behavioral and fMRI work.	Deep-target access and potential focality advantages depending on skull transmission and acoustic parameters.	State dependence and bidirectional effects; need sham and off-target controls and explicit artifact management for EEG.	Deffieux et al., 2013; Yang et al., 2021; Klink et al., 2021; Mohammadjavadi et al., 2021; Gaur et al., 2020; Legon et al., 2018.

In the subsections below, we summarize (i) transcranial magnetic stimulation, (ii) transcranial electrical stimulation (tDCS and tACS), (iii) temporal interference stimulation, and (iv) transcranial focused ultrasound, (also see Figure 1B, right) emphasizing what each approach can modulate, what can be measured reliably in primates, and what mechanistic and translational questions are realistic in current NHP workflows.

### 6.1 Transcranial magnetic stimulation (TMS) and EEG in NHPs

Transcranial magnetic stimulation uses brief, strong magnetic field pulses to induce currents in the cortex, and in humans TMS combined with EEG can index cortical reactivity and connectivity. In NHPs, TMS is particularly useful for two complementary objectives: (1) single-pulse paradigms that quantify short-latency TMS-evoked responses and their propagation across connected regions as an assay of effective connectivity, and (2) repetitive paradigms that test protocol-specific changes in oscillatory activity and longer-lasting aftereffects. This framing helps distinguish perturbation-evoked connectivity questions from entrainment and aftereffect questions when interpreting stimulation effects in aging and disease models.

In NHPs, TMS has been employed both to perturb behavior and to directly record neural responses. One advantage of NHP models is the ability to use invasive electrodes alongside TMS to bypass the muscle artifacts that plague human TMS-EEG studies (Perera et al., 2023). For instance, a study delivered single-pulse TMS to awake monkeys while recording intracortical EEG and deep LFPs; this approach dissociated the direct cortical response from indirect peripheral effects such as evoked muscle twitches (Romero et al., 2019). The findings indicated that TMS can trigger a short-latency excitation (about 10 ms) in the targeted cortex followed by a brief suppression, and that these TMS-evoked potentials propagate to connected areas, supporting effective connectivity mapping (Romero et al., 2019). At the single-neuron level, recordings in monkeys have shown that TMS causes a transient burst of spiking in a localized patch of cortex, consistent with focality of the induced electric field at the neuronal scale (Romero et al., 2019). Task context can also influence stimulation effects in awake primates, including task-related variation in corticospinal output evoked by TMS (Baker et al., 1995), which reinforces the value of reporting behavioral state and task epoch when interpreting evoked responses.

Combining TMS with surface EEG in monkeys is more challenging due to size and coil positioning, but some experiments have succeeded in evoking EEG-measurable responses. To mitigate artifacts, one group employed specialized EEG electrodes and post-pulse subtraction techniques in anesthetized macaques, demonstrating that low-frequency rTMS (1 Hz) produced a lasting increase in delta-wave power, akin to inducing a drowsy cortical state. Because vigilance state can shape evoked responses, it is useful to report state monitoring explicitly; for example, cortical evoked responses to magnetic stimulation in macaque were reported during slow-wave sleep but not during wakefulness or REM sleep (Pigarev et al., 2008). Complementary mechanistic evidence in primates also comes from molecular imaging: in anesthetized macaques, rTMS over primary motor cortex has been linked to endogenous dopamine release measured with [11C]raclopride PET (Ohnishi et al., 2004).

Additionally, repetitive TMS protocols (like theta-burst stimulation, TBS) have been tested in monkeys to probe plasticity. Continuous TBS applied over parietal cortex in macaques led to both behavioral changes (biases in visuomotor tasks) and underlying neural changes observable in local field potential spectra. These results paralleled human cTBS effects and provided causal validation that TMS-induced oscillatory changes correspond to functional impairments (Mitoma et al., 2022). Notably, primate cTBS work also provides a concrete way to separate immediate evoked effects from longer-lasting changes in excitability by probing post-TBS responses with single-pulse TMS (Romero et al., 2022).

Disease-model use cases are emerging in primates, but several foundational parkinsonian primate datasets involve chronic epidural motor cortex stimulation rather than transcranial magnetic stimulation. In MPTP-treated primate models, motor cortex stimulation has been reported to yield behavioral benefit and electrophysiological changes consistent with reduced pathological basal ganglia activity patterns (Drouot et al., 2004), while other studies report mild and transient benefit depending on stimulation settings and context (Wu et al., 2007). Although these studies are not TMS *per se*, they provide primate evidence that modulating motor cortical circuits can influence downstream basal ganglia physiology and behavior, supporting the rationale for TMS protocols that aim to shift evoked propagation and oscillatory state in parkinsonian networks.

Moving forward, TMS-EEG in NHPs will be instrumental for understanding the mechanisms of TMS by directly visualizing how a TMS pulse perturbs ongoing oscillations or entrains new ones and for optimizing stimulation parameters before trials in humans. It also contributes to safety evaluations (ensuring TMS does not cause pathological spikes in primate brains). With improvements in coil design (e.g., focal coils for monkeys) and artifact reduction, TMS-EEG could become a routine paradigm in cognitive neuroscience experiments on NHPs. Across primate TMS studies, interpretability improves when authors separate early evoked components from later sensory confounds, state whether readouts are scalp EEG versus invasive electrophysiology, and report whether effects are observed during stimulation, immediately after, or as delayed aftereffects.

### 6.2 Transcranial electrical stimulation (tDCS/tACS) and EEG in NHPs

Transcranial direct current stimulation (tDCS) and alternating current stimulation (tACS) are attractive for modulating brain activity with a simple electrode setup. In NHPs, a key advantage is that electrical stimulation can be paired with concurrent invasive recordings (single units, LFP, ECoG) and scalp EEG, providing a direct link between montage and dose and circuit-level effects.

Evidence from NHP studies corroborates many findings from human tDCS/tACS research and offers deeper insight via concurrent electrophysiology. For example, applying tDCS to the prefrontal cortex of rhesus monkeys was found to facilitate cognitive performance in one experiment: anodal tDCS over the dorsolateral prefrontal area significantly improved the monkeys’ accuracy and learning speed in an associative memory task (Fehring et al., 2024). This aligns with human data where prefrontal tDCS can enhance working memory or learning, and it validates the approach in a controlled setting (Assecondi et al., 2021; Pergher et al., 2022).

On the neural level, invasive recordings during and after tDCS in monkeys have shown that DC stimulation induces polarity-specific shifts in cortical excitability. At the single-cell level, anodal tDCS tends to increase the firing rates of neurons and facilitate long-term potentiation-like effects, whereas cathodal can suppress firing and induce LTD-like effects (Fehring et al., 2024). Interestingly, one study reported that tDCS not only modulated neuron firing but also decoupled the normal relationship between neural firing rate and task performance, suggesting a complex neuromodulatory impact (Fehring et al., 2024). This motivates reporting both behavioral endpoints and neural endpoints within the same animals, since stimulation may change performance through mechanisms not fully captured by mean firing-rate changes alone.

Transcranial alternating current stimulation (alternating currents) offers frequency-specific intervention, and primate experiments have leveraged this to target particular brain rhythms (Krause et al., 2019). In marmosets, for instance, transcranial 8 Hz stimulation (alpha-band) was applied while recording EEG and it entrained an enhanced alpha oscillation in occipital cortex, temporarily altering visual processing (Johnson et al., 2020; Vieira et al., 2025). When using tACS with EEG readouts, it is useful to distinguish effects measured during stimulation (where stimulation artifact can dominate) from post-stimulation aftereffects (where standard spectral and ERP analyses can be applied more directly).

High-definition tDCS/tACS, using smaller electrodes in arrays, has also been implemented in macaques combined with imaging: a recent approach delivered HD-tDCS to one hemisphere while acquiring fMRI, showing widespread network connectivity changes and confirming current flow models (Hoffner et al., 2024). Such combined stimulation-imaging designs are useful for bridging “local dose at the electrode” to “network-level consequence,” which remains a recurring interpretation challenge for transcranial electrical stimulation.

From a safety perspective, NHP studies help establish safe intensity limits and examine any histological changes from repeated sessions. So far, low-intensity tDCS/tACS appears well-tolerated by monkeys, with no behavioral distress and only mild, transient EEG changes during stimulation (Hoffner et al., 2024). Where invasive recordings are available, safety reporting can be strengthened by explicitly stating whether stimulation induced seizure-like firing or abnormal discharges, alongside behavioral tolerance and post-session neurological assessment.

This paves the way for using tDCS or tACS chronically in NHP models of disease, for example, applying theta-frequency tACS to an NHP model of Alzheimer’s disease to test whether memory-related theta oscillations and cognition improve. In summary, transcranial electrical stimulation in primates, monitored by behavioral tests and EEG/LFP recordings, is a valuable translational step for neuromodulation therapies. Key methodological gaps remain in establishing how montage, electrode size, and brain state (wake, sleep, anesthesia) shape the delivered electric field and the direction of physiological effects, so future NHP studies should prioritize field modeling, within-subject sham controls, and explicit state monitoring to improve comparability across studies.

### 6.3 Temporal interference (TI) stimulation and EEG in NHPs

Temporal interference stimulation is a novel technique aiming for non-invasive deep brain stimulation by overlapping two high-frequency currents that interfere to produce a lower-frequency envelope in targeted deep tissue (Grossman et al., 2017). After initial validation in rodents (Grossman et al., 2017), TI stimulation is being explored in primates to evaluate its focality and effects on neural activity.

A complementary line of evidence uses modeling and neuroimaging to assess whether TI can reach deep targets in primates and how focality scales with brain size and electrode montage (Liu et al., 2024). Because envelope maxima can occur in cortex as well as at depth, primate studies benefit from explicitly testing “on-target” versus “off-target” physiological signatures using multi-site recordings, rather than inferring target engagement from montage alone (Liu et al., 2024).

Recent work by Vieira et al. (2024) provided the first direct neurophysiological evidence of TI effects in macaques. In their setup, two alternating currents 2 kHz each were applied with a slight frequency difference e.g., 2000 and 2020 Hz such that an amplitude-modulated envelope at 20 Hz formed in a specific cortical region (Vieira et al., 2024). While delivering TI-tACS, they recorded single-unit spikes and local field potentials from electrodes near the interference focus. The results showed that TI stimulation did not drive overall firing rate changes but did modulate the timing of neuronal spikes relative to the 20 Hz envelope. In some neurons, TI-tACS entrained them to fire consistently at a particular phase of the 20 Hz oscillation increasing the phase-locking value from near 0 to 0.17. In other neurons, especially those that had an intrinsic rhythmic firing at baseline, TI-tACS disrupted their regularity significantly decreasing their phase-locking. Importantly, across 234 neurons sampled in multiple cortical areas, around 28 showed significant changes in spike timing due to TI stimulation, predominantly reductions in their endogenous rhythmicity. These findings confirm that TI can penetrate the primate skull and modulate neural activity in a behaviorally relevant frequency range, supporting its potential for non-invasive deep neuromodulation (Liu et al., 2024; Vieira et al., 2024).

Mechanistically, TI remains debated, and biophysical work argues that TI effects cannot be explained by passive membrane filtering alone, proposing ion-channel mediated rectification mechanisms and motivating explicit controls and careful reporting of stimulation parameters and physiological validation (Mirzakhalili et al., 2020). Ongoing studies are extending TI to target deeper structures like the primate hippocampus or basal ganglia, with simultaneous EEG or ECoG recording to see if specific brain rhythms e.g., theta in hippocampus can be enhanced or suppressed (Kim et al., 2023; Violante et al., 2023).

Bridging to humans, TI targeting of the hippocampus has been supported by electric-field modeling and intracranial measurements in a cadaver, together with fMRI and behavioral results consistent with hippocampal engagement and episodic memory modulation (Violante et al., 2023).

If TI can be refined to reliably affect deep brain oscillations without affecting cortex above, it could be a breakthrough for treating disorders like Parkinson’s or epilepsy non-invasively. In the meantime, NHP experiments are crucial for mapping the interference patterns and verifying via EEG LFP that the intended modulation frequency appears at the target and not elsewhere. This validation step is particularly important because stimulation artifacts are strongest at the carrier frequencies, while biological effects are expected at the envelope frequency, motivating careful separation of stimulation-period signals from post-stimulation aftereffects in EEG analyses. So far, computational models of TI in a rhesus monkey head suggest the method can focus energy at a 1–2 cm region in the brain (Botzanowski et al., 2023; Yatsuda et al., 2024). Combining those models with empirical EEG recordings in monkeys for instance, looking for evoked oscillations on scalp EEG when TI is applied will further validate the technique.

Bridging more broadly to humans, TI has been reported to modulate motor-network measures, including increased functional connectivity between primary motor cortex and secondary motor regions during stimulation (Zhu et al., 2022). Human deep-target studies further indicate that TI can engage the striatum, with theta-burst patterned striatal TI linked to increased striatal and motor-network activity and improved motor skill learning (Wessel et al., 2023) and high-gamma striatal TI linked to disruption of reinforcement-related components of motor skill learning with corresponding fMRI effects (Vassiliadis et al., 2024). These human studies strengthen the translational bridge but also reinforce the need for harmonized target-engagement criteria and reporting standards that allow quantitative comparison across species and laboratories.

In summary, temporal-interference stimulation is an exciting frontier, and NHP research is confirming its ability to shift neural timing without surgical implants.

### 6.4 Transcranial focused ultrasound (tFUS) neuromodulation and EEG in NHPs

Low-intensity transcranial focused ultrasound (tFUS) can modulate neural activity through mechanical effects including pressure wave induced perturbation of membrane and channel dynamics. In primates, tFUS is appealing because it can reach deep targets that are difficult to access with surface electrical stimulation or TMS, and its effective focality can be higher than many non-invasive techniques, although focal volume depends on skull transmission and acoustic parameters. Evidence in non-human primates supports both behavioral and network-level effects. Deffieux et al. (2013) showed that low-intensity focused ultrasound can modulate monkey visuomotor behavior, including reversible changes in oculomotor choice behavior when targeting frontal oculomotor circuitry. In addition, MRI-guided primate studies report state-dependent and bidirectional modulation of brain activity by tFUS, reinforcing that ongoing brain state (awake, anesthetized) can shape the direction of the net effect (Yang et al., 2021). Conversely, ultrasound can also have inhibitory or disruptive effects. A notable study applied tFUS to the thalamus (pulvinar) of monkeys during a visual discrimination task and reported impaired detection performance during sonication, consistent with transient disruption of a subcortical relay (Klink et al., 2021; Yu and He, 2024).

Electroencephalography is a natural candidate readout for scaling primate tFUS studies toward longitudinal designs in aging and neurodegeneration, but robust NHP tFUS-EEG evidence remains comparatively limited relative to behavior and fMRI. Methodologically, a related large-animal study (sheep) demonstrated that MRI-guided ultrasound targeting of the lateral geniculate nucleus can reversibly suppress visual evoked potentials and alter time-frequency dynamics, while also quantifying electromagnetic coupling artifacts and mitigation strategies that are directly relevant when co-registering ultrasound hardware with EEG electrodes (Mohammadjavadi et al., 2021). In primates, adopting the same logic is helpful: include sham and off-target controls, explicitly monitor vigilance state, and separate stimulation timing from analysis epochs to limit ultrasound-related artifacts in EEG features.

Safety statements should be anchored to primate histology whenever possible. For example, Gaur et al. (2020) reported histologic safety assessments in rhesus macaques (alongside sheep) after transcranial ultrasound neuromodulation protocols, supporting continued primate work under defined parameter regimes and reporting standards.

Bridging to humans, transcranial ultrasound studies targeting thalamic circuitry have reported modulation of somatosensory processing and evoked responses, providing a translational benchmark for the kinds of EEG endpoints that primate tFUS experiments could aim to normalize or shift under explicit state control (Legon et al., 2018).

The future will likely see combined tFUS-EEG paradigms to explore therapeutic stimulation in NHP disease models, for example testing whether repeated tFUS to basal forebrain shifts EEG markers of arousal and memory in aged monkeys, or whether tFUS targeting cortico-basal ganglia nodes reduces pathological beta-band signatures in parkinsonian monkeys. To increase interpretability across laboratories and species, these experiments benefit from predefined EEG endpoints (evoked potential amplitude and latency, band-limited power and burst statistics, and connectivity measures) plus within-subject sham and off-target controls.

## 7 Ethical and translational considerations

### 7.1 Animal welfare and research ethics in primate EEG studies

Electroencephalography research involving non-human primates requires stringent ethical governance, grounded in recognition of their advanced cognitive capacities and ability to experience distress (Ramos et al., 2023). Such studies are conducted under regulatory frameworks and institutional review boards that scrutinize experimental objectives against potential risks to animal welfare (Ramos et al., 2023). Central to this oversight is the principle of the 3Rs: Replacement, Reduction, and Refinement (Graham and Prescott, 2015). In addition to the 3Rs, ethical review rests on an explicit harm-benefit analysis and a clear justification for species choice, particularly for invasive procedures. In this review’s context, primate EEG studies are most defensible when the scientific question depends on primate-specific neuroanatomy, network organization, or cognitive behavior that cannot be addressed adequately in other model systems, and when the goal is translation to humans using comparable EEG endpoints.

In practice, Replacement entails prioritizing non-invasive imaging methods or computational simulations when they can address the scientific question. Reduction is achieved through optimized study design, statistical power analysis, and data-sharing consortia to avoid redundant experiments. Refinement focuses on minimizing discomfort, for instance by employing positive reinforcement training so that animals voluntarily accept EEG cap placement or positioning in a primate chair, thereby substantially reducing stress (Nakamura et al., 2020).

Non-invasive EEG procedures are inherently painless. When invasive recordings such as electrocorticography require surgical implantation, these are carried out under deep anesthesia, followed by multimodal analgesic protocols during recovery. Contemporary primate facilities maintain enriched living environments by incorporating social housing, foraging puzzles, and manipulable objects to safeguard psychological health. Continuous veterinary monitoring is standard; any skin irritation from EEG caps is promptly addressed, whether through temporary removal, topical treatment, or adjustments in fit. Experiments are paused or terminated at the first indication of undue harm. In studies of aging or neurodegenerative disease, predefined humane endpoints are established, ensuring that if clinical decline surpasses set thresholds, euthanasia is carried out humanely (Ramos et al., 2023).

Given the rarity and high value of NHP subjects, there is an ethical imperative to maximize data yield per individual (Graham and Prescott, 2015). This often involves multipurpose protocols where a single subject contributes EEG, MRI, and behavioral datasets across research aims, as well as open-access dissemination of datasets to avoid repetitive use of new animals. Ethical review processes increasingly assess emerging alternatives, such as human brain organoid models or advanced computational simulations, which could replace certain NHP experiments in the future. Transparency in communicating the rationale for NHP research and its welfare safeguards to the public remains essential. Ongoing dialogue within the neuroscience community underscores that primate experiments proceed only when no other model can adequately address the scientific objectives, and every measure is taken to minimize harm while preserving scientific integrity. This ethical infrastructure sustains both the moral and scientific legitimacy of primate EEG research (Ramos et al., 2023).

### 7.2 Translational implications for human aging and neurodegenerative disease

Insights from primate EEG studies have direct implications for human health. Because NHPs so closely model the human brain’s complexity, findings in these animals often accelerate clinical translation. This translation is most effective when primate and human studies share the same operational EEG endpoints and behavioral constructs, enabling bidirectional hypothesis testing across species. For instance, identifying an EEG biomarker of early cognitive decline in a NHP can inform clinicians what to look for in middle-aged humans at risk of dementia (Merkin et al., 2023). Indeed, one motivation for primate studies is to establish biomarkers that are translatable, using the same EEG technology in monkeys and humans to ensure the marker is comparable. Evoked EEG paradigms are particularly well suited for this goal because their stimulus timing reduces ambiguity about vigilance state and supports harmonized analysis pipelines across laboratories (Tada et al., 2020; Nakamura et al., 2022). As mentioned, changes like increased theta oscillations or epileptiform spikes found in an Alzheimer’s-like monkey suggest that similar EEG monitoring in elderly people could detect prodromal AD and prompt early intervention (Haberman et al., 2017; Yu T. et al., 2021). However, mechanistic correspondence cannot be assumed: similar spectral changes may reflect distinct drivers across species (for example, hyperexcitability, altered inhibition, or changes in arousal regulation), so translational EEG biomarkers benefit from being anchored to convergent readouts such as imaging, CSF biomarkers, or (in primates) invasive LFP validation (Wong et al., 2022). Moreover, primate research helps to validate targets for intervention. If enhancing a certain brain rhythm (say gamma oscillations) via neuromodulation improves memory in an NHP model that provides a strong rationale to trial a gamma stimulation therapy in human patients. Similarly, NHP work can refine stimulation parameters by linking dose and montage to neural engagement at the single-unit and LFP level, thereby constraining what “target engagement” should look like in human EEG (Krause et al., 2019).

The translational pathway is evident in neuromodulation techniques: repetitive TMS protocols that showed cognitive benefits in aged monkeys are now being tested in clinical trials for mild cognitive impairment (Drumond Marra et al., 2015; Yan Y. et al., 2023; Vieira et al., 2025). In parallel, parkinsonian NHP models provide a direct bridge between circuit pathology and scalable EEG endpoints, because pathological beta synchronization and beta burst abnormalities can be measured with invasive basal ganglia LFPs and related to scalp and ECoG signatures, supporting endpoint selection for human trials (Connolly et al., 2015; Vieira et al., 2025). Likewise, safety and dosing for novel methods like temporal interference stimulation will be determined in monkeys first, de-risking subsequent human applications. This is particularly relevant for methods where the expected biological effect occurs at a low-frequency envelope while the stimulation artifact is concentrated at much higher carrier frequencies, making primate validation essential for separating physiological signals from artifactual contamination (Mirzakhalili et al., 2020; Liu et al., 2024; Vieira et al., 2024).

Another area is drug development where EEG in NHPs can be used to evaluate pharmacological treatments. For example, a new dopamine agonist for Parkinson’s might be given to MPTP-treated monkeys; if their EEG beta abnormalities normalize and behavior improves, it bolsters confidence for human trials. Because these models also allow within-subject longitudinal tracking, EEG can serve as a pharmacodynamic readout that complements behavioral endpoints and reduces reliance on terminal histology for iterative compound screening (AlShimemeri et al., 2021; Davin et al., 2022).

The high correspondence between primate and human EEG patterns (e.g., monkeys exhibit a 40 Hz auditory steady-state response similar to humans) means that therapeutic effects on EEG in monkeys are likely predictive of human outcomes (Farmer, 1998; Criscuolo et al., 2023). That said, cross-species comparability is strongest when recordings are matched for behavioral state and preprocessing choices (referencing, artifact removal, and spectral parameterization), which can otherwise shift band-power estimates and inflate apparent agreement (Nakamura et al., 2020; Wong et al., 2022).

Beyond specific diseases, primate EEG studies contribute to basic knowledge about the aging brain that is hard to obtain in humans (because invasive measures or controlled longitudinal studies are impractical). This knowledge can inform lifestyle or clinical recommendations; for instance, if enriched environments are shown via EEG to preserve frontal lobe function in aging monkeys, it underscores the importance of mental stimulation in human aging (Lacreuse et al., 2020; Xu et al., 2025). Importantly, these datasets can help distinguish intervention-related preservation of network dynamics from non-specific changes driven by altered sleep, stress, or physical activity, all of which can confound human observational studies (Zhdanova et al., 2011; Nakamura et al., 2020). It’s also worth noting that primate neuroimaging and neurophysiology research has a feedback loop with human studies; hypotheses often flow from clinical observations to the lab and back. NHP work on Alzheimer’s and Parkinson’s is done with the explicit goal of improving diagnosis and treatment of those human diseases. As such, translational neuroscience frameworks increasingly involve NHPs as a necessary step after rodents before human trials (Milham et al., 2020). In practical terms, NHP studies can be used to pre-register electrophysiological endpoints and analysis pipelines before launching human trials, reducing flexibility in biomarker definition and increasing the interpretability of negative results.

The continued refinement of primate EEG and multimodal techniques will ensure that the data we get is as relevant as possible, measuring the same signals and endpoints that matter in the clinic. In summary, the translational impact of primate EEG research is substantial; it validates EEG biomarkers, tests causal interventions on brain rhythms, and ultimately helps bridge the gap between laboratory discoveries and therapies that improve the lives of patients with age-related neurological disorders. Future progress will depend on multicenter harmonization of acquisition and preprocessing, explicit state monitoring, and combined invasive-to-non-invasive validation studies that define which primate EEG features are most predictive of human clinical outcomes.

## 8 Conclusion and future directions

Electroencephalography in non-human primates is an increasingly practical and informative approach to study brain dynamics of aging and neurodegeneration. Across the studies reviewed here, the strongest translational value comes from (i) using EEG endpoints that match human clinical readouts and (ii) leveraging primate-only capabilities, including controlled longitudinal sampling, invasive validation, and causal perturbations. Through recent advances in methodology, from non-invasive caps and active electrodes to wireless systems, researchers can record high-quality EEG from awake monkeys, even over long periods and during complex behaviors. Because EEG phenotypes depend strongly on behavioral state and artifact burden, future work should report vigilance state, muscle contamination control, and referencing choices in a harmonized manner to support quantitative cross-study comparison. These technical gains are yielding new scientific insights. We now recognize that normal aging in primates is accompanied by specific EEG changes (e.g., slowed oscillations and network hyper-synchrony), and that primate models of Alzheimer’s and Parkinson’s exhibit EEG biomarkers closely paralleling human patients (such as EEG slowing in AD or exaggerated beta rhythms in PD). However, mechanistic correspondence should be tested rather than assumed, ideally by linking scalp EEG features to invasive LFP or ECoG recordings and to independently measured disease-relevant outcomes (behavioral performance and molecular or imaging markers when available). By integrating EEG with other modalities like MEG, fMRI, and invasive electrophysiology, we achieve a multi-scale understanding of how neural circuits deteriorate or compensate with age. Furthermore, leveraging EEG as both a readout and feedback control for neuromodulation has opened causal tests of candidate rhythms and circuits in NHPs. Techniques like TMS, tDCS and tACS, temporal interference, and focused ultrasound, tested in primates with EEG guidance, can constrain stimulation parameters and target-engagement criteria before translation to human studies.

Looking ahead, several future directions are clear: First, improve comparability across laboratories by adopting shared acquisition and preprocessing standards for NHP EEG, including explicit state monitoring and predefined primary EEG endpoints (spectral slowing indices, burst statistics, and evoked-response measures). Second, increase mechanistic anchoring of candidate biomarkers by pairing scalp EEG with invasive recordings in the same animals, enabling frequency-resolved “ground-truth” mappings that clarify which circuit events are detectable at the scalp and under what conditions. Third, expand longitudinal designs in aging and disease models to connect early EEG changes to later cognitive decline and neuropathology, aligning primate staging with human trial-relevant endpoints. Fourth, develop closed-loop intervention studies in NHPs where EEG or invasive signals define target engagement and trigger stimulation, allowing direct tests of whether normalizing a network signature produces durable behavioral benefit and whether the same signature can serve as a clinical endpoint in humans. Finally, the continued emphasis on refining animal welfare and ethical research practices will ensure that these scientific advances proceed responsibly. In conclusion, primate EEG provides a direct bridge between mechanistic circuit neuroscience and human-scalable biomarkers. It offers a pathway to discover early diagnostic signals and test interventions in a setting that closely mirrors the human condition. The most actionable next step is to link specific EEG features to specific mechanisms and outcomes in NHPs, then carry those same endpoints into human observational studies and interventional trials.
